# A Review on Current Strategies for Extraction and Purification of Hyaluronic Acid

**DOI:** 10.3390/ijms23116038

**Published:** 2022-05-27

**Authors:** Carlos Dariel Rodriguez-Marquez, Susana Arteaga-Marin, Andrea Rivas-Sánchez, Renata Autrique-Hernández, Roberto Castro-Muñoz

**Affiliations:** 1 Tecnologico de Monterrey, Campus Chihuahua, Avenida H. Colegio Militar 4700, Nombre de Dios, Chihuahua 31300, Chihuahua, Mexico; carlosdarielrm10@gmail.com; 2 Tecnologico de Monterrey, Campus Querétaro, Avenida Epigmenio González 500, San Pablo, Santiago de Querétaro 76130, Qro., Mexico; susartm@gmail.com (S.A.-M.); renautriq@gmail.com (R.A.-H.); 3 Tecnologico de Monterrey, Campus Monterrey, Avenida Eugenio Garza Sada 2501 Sur, Tecnológico, Monterrey 64849, N.L., Mexico; riv.andreafernanda@gmail.com; 4 Tecnologico de Monterrey, Campus Toluca, Avenida Eduardo Monroy Cárdenas 2000 San Antonio Buenavista, Toluca de Lerdo 50110, Mexico; 5 Department of Process Engineering and Chemical Technology, Faculty of Chemistry, Gdansk University of Technology, 11/12 Narutowicza St., 80-233 Gdansk, Poland

**Keywords:** hyaluronic acid (HA), glycosaminoglycan, fermentation, precipitation, bacterial production, extraction, microfiltration (MF), ultrafiltration (UF), diafiltration (DF)

## Abstract

Since it is known that hyaluronic acid contributes to soft tissue growth, elasticity, and scar reduction, different strategies of producing HA have been explored in order to satisfy the current demand of HA in pharmaceutical products and formulations. The current interest deals with production via bacterial and yeast fermentation and extraction from animal sources; however, the main challenge is the right extraction technique and strategy since the original sources (e.g., fermentation broth) represent a complex system containing a number of components and solutes, which complicates the achievement of high extraction rates and purity. This review sheds light on the main pathways for the production of HA, advantages, and disadvantages, along with the current efforts in extracting and purifying this high-added-value molecule from different sources. Particular emphasis has been placed on specific case studies attempting production and successful recovery. For such works, full details are given together with their relevant outcomes.

## 1. Introduction

Skin aging is a complex biological process related to two independent mechanisms: intrinsic and extrinsic. The first one is not preventable and occurs naturally in all human beings. It is influenced by hormonal changes due to aging and is also strongly related to the reduction of cells in the basal layer and skin moisture problems. On the other hand, the second mechanism is the result of exposure to external environmental factors, such as solar ultraviolet radiation (UV), lack of essential nutrients, air pollution, and skin moisture [1]. In both mechanisms, skin hydration plays an important role in skin aging since proper hydration contributes to becoming plump and improves skin elasticity; therefore, the majority of conventional treatments focus on this. These treatments are soft tissue augmentation products, commonly called “fillers.” These fillers have become very popular in the beauty–cosmetic industry since they use botulinum toxin type A, colloquially known as Botox, for the treatment and correction of facial expression lines in the upper third of the face [2]. 

One of the main causes of premature skin aging is the frequent and extended exposure to UV radiation. This exposure causes damage as an initial mild form of wound healing, and this phenomenon is related to an increase in dermal HA, indicating that UV radiation induces a notorious damage in skin. UV exposure contributes to approximately 80% of premature facial skin aging [3]. 

**Figure 1:**
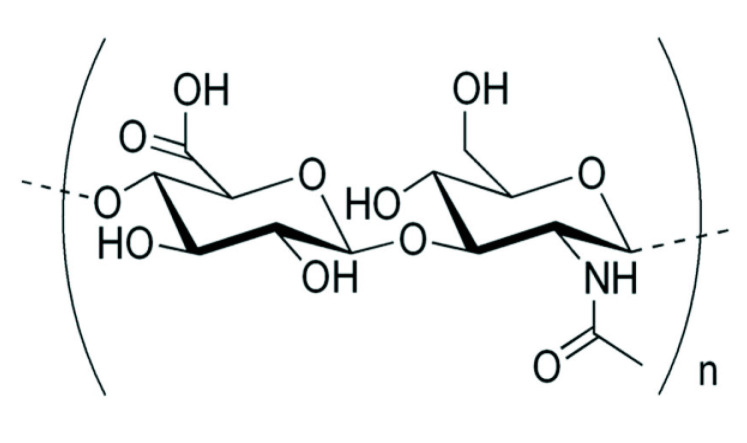
Chemical structure of a hyaluronic acid molecule [4].

In recent years, a key molecule has emerged as a popular and novel solution to the problem of skin moisture: hyaluronic acid (HA). HA, also known as hyaluronan, is a glycosaminoglycan composed of repeating polymeric disaccharides of uronic sugar (D-glucuronic acid) and amino sugar (N-acetyl-D-glucosamine), both linked by glucoronidic β, 1–3 glycosidic bonds, as illustrated in Figure 1. 

HA is naturally produced by both eukaryotic and prokaryotic cells, but it has not been found in fungi, plants, or insects [3,5]. In the human body, the total content of HA is about 15 g for a 70 kg adult, and approximately 50% of this HA is present in the skin (dermis and epidermis). It is mainly produced by cells such as fibroblasts, synoviocytes, and chondrocytes. HA has a naturally negative charge due to carboxylate groups. This negativity forms highly hydrophilic salts that have the special and unique capacity of binding and retaining water molecules (H_2_O), allowing for excellent viscoelasticity, high biocompatibility, high moisture retention capacity, and hygroscopic properties. These properties make HA act as a water balance retention–flow-resistant regulator, lubricant, space filler, and shock absorber [3,4].

HA molecules have different physico-chemical properties; for instance, in aqueous solutions, they form a stable β-sheet tertiary structure due to the intermolecular hydrogen bonds and hydrophobic interactions, enabling the formation of an extended meshwork that depends on HA molecular weight (MW) and concentration. The vast number of configurations, shapes, and rheological properties of HA molecules directly depend on the salt concentration, size, ionic strength, pH, and temperature [3,5]. The volume of HA molecules increases with a higher MW of the polymer. That is, that if the length of the polymer increases, the density decreases because the increase in mass is slower than that of the volume. In other words, HA with a high molecular weight occupies a large volume in space. This latter property makes HA polymers commonly used as space fillers [5,6]. 

HA has been deeply studied since the last century. Most studies recognize that HA plays an important role in a large number of physiological, pathological, and biological processes, but one key factor for these actions is its MW. HA’s molecular weight directly depends on the extraction source (see Table 1); if HA is extracted from an animal, the MW will be large (up to 20,000 kDa), which is much larger than that extracted from bacteria or yeast (1000–4000 kDa). This difference in MW could determine the biological action of the molecule [6]. HA with HMW is used in products for orthopedics, cosmetics, ophthalmology, and tissue engineering. In contrast, HA with a low molecular weight (LMW) is useful for the production of products that inhibit tumor progression and stimulate the production of proinflammatory cytokines, chemokines, and growth factors [7]. 

HA is a molecule with many benefits for humans, but recently the cosmetic industry has focused on creating products with HMW HA, using its hydration, lubrication of joints, and space-filling capacity for skin care. Progressive reduction in the size of HA polymers in the epidermal skin has been reported to be affected by aging; however, at the same time, skin aging is caused by the lack or reduction of HA molecules in the skin, so this relation between HA and skin aging becomes a vicious circle, where the lack of HA causes skin-aging problems, and at the same time, skin aging induces a HA level decrease.

**Figure 2:**
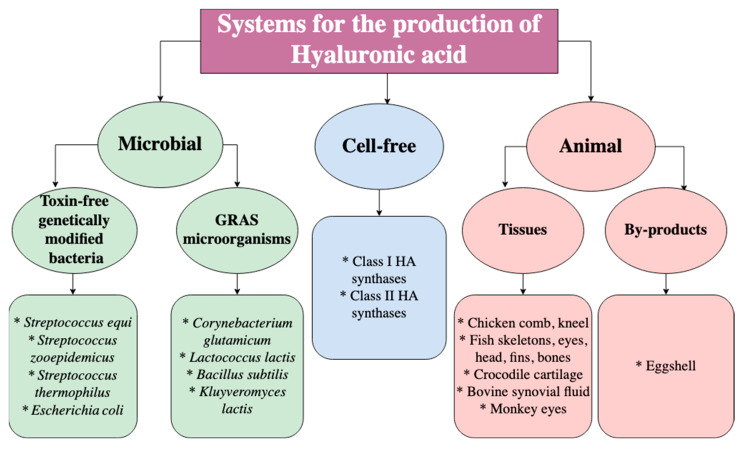
Different systems for the production of HA [8].

As mentioned before, HA can be obtained from different sources, including animal tissues, microorganisms, and cell-free systems such as enzymes, as shown in Figure 2. Importantly, the industrial production of HA is based on animal tissues since it is present in all vertebrates and certain parts of the animal body that contain a high concentration of HA, i.e., skin, eyes, synovial fluid between joints, umbilical cord, etc. [8]. Furthermore, these animal tissues can be acquired from animal waste and by-products that would otherwise be discarded, leading to economic and environmental benefits [9]. Over the last few decades, industrial manufacturing of HA has also been based on microorganisms due to extensive research in bacterial fermentation. Interestingly, bacteria produce HA that is identical to that from animal sources; therefore, it does not trigger immune responses and can be used in medical-grade products or for cosmetic purposes. Several bacterial species can be employed to produce HA, including genetically engineered strains and GRAS (generally recognized as safe) bacteria [8]. Unfortunately, there are limitations to animal and bacterial production related to purity and contamination, in which the production in vitro can be overcome through the use of enzymes from Class I HAS and Class II HAS family members. However, these cell-free systems are still not optimized since they display very low yields and do not represent an alternative for industrial production [7].

It is worth mentioning that the importance of HA has increased because of its good biocompatibility and positive results. To some extent, it is close to being considered the ideal filler in the beauty–cosmetic industry. Safe consumption is also important; therefore, the ideal filler must be meet various relevant requirements in terms of being non-carcinogenic, non-allergenic, non-teratogenic, and free from all transmittable diseases for users [2]. Another important part of the safety of products containing hyaluronan is the molecular weight of this biomolecule either for medical, pharmacology, or cosmetic purposes. The required molecular weight may vary depending on the application (see Table 2).

There are many methods for the isolation and purification of HA. To select an isolation method, it is important to maintain the intrinsic properties of polysaccharides during the process. In recent years, various methods have been studied, especially hot water extraction, which is widely used because of the principle that most polysaccharides have higher solubility in water, and are also more stable in hot water [19]. Another method is digestion using enzymes. HA extraction methods present advantages and disadvantages when considering cost, degree of purification, and environmental impact. Generally, the lower-cost methods yield a lower purity in comparison with the high-purity methods, which require more steps and larger amount of reagents. In comparison with the previous two methods mentioned before, the use of enzymes is expensive and time consuming, a significant number of reagents is required to hydrolyze the tissue, and additional heat treatment is required to stop the hydrolysis process. On the other hand, the use of organic solvents is cheaper and does not require the use of enzymes or heat treatment in any step, it is easier to perform, and it is less time consuming. Nowadays, the optimization of current extraction methods is important to ensure an efficient isolation at high purity, implying a low-cost, less time-consuming, and environmentally safe technique [9].

The main challenge of HA is not its production or effectiveness, but its extraction and purification processes, since effective and low-cost strategies are needed in order to obtain HA with the necessary requirements, such as high quality and purity, high production yield, HMW, uniformity, etc. Therefore, the present review is focused on the analysis of the current strategies for the production of HA at the research level, pointing out the current progress in the field. A particular emphasis has been devoted to relevant development works and related outcomes. The present review also presents the most innovative, efficient, and profitable strategies for the extraction and purification of HA. 

## 2. Advances in HA Bacterial Production, Extraction, and Purification

As mentioned previously, HA is naturally produced by different mechanisms depending on the sources. Most of the HA used in current industries is obtained from animal tissues and microbial fermentation. Nowadays, industries are looking for more economically profitable production options with controlled parameters, high yield of production, rentable production costs, uniformity of product, and HA with a HMW and purity. At this point, HA bacterial production, extraction, and purification represents the ideal pathway for biomedical purposes or products. However, within this natural production strategy exists the risk of contamination with bacterial endotoxins, nucleic acids, proteins, and heavy metals. This has led to the use of endotoxin-free microorganisms that are genetically modified to express the genes involved in HA synthesis in order to obtain HA with HMW and high purity or modified to avoid the presence of toxins in the media [8]. 

**Figure 3:**
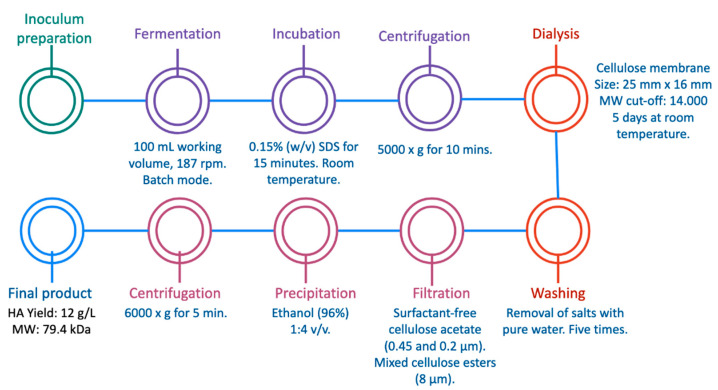
Hyaluronic acid production by *Streptococcus equi* ssp. *equi* by a sequential process [20].

Over the last two decades, many authors have researched the most profitable and effective bioprocess for the obtention of high-quality and HMW hyaluronic acid in order to scale up the bioprocess for industrial purposes. Different microorganisms have been used as HA producers, varying different fermentation methods and culture media parameters, but there is still a research gap for better production and extraction conditions to obtain more purified HA with high-yield results. For instance, Güngor et al. recently conducted a study in which HA was produced at a laboratory scale by fermentation of *Streptococcus equi* ssp. *equi* (see Table 3) with the aim of establishing an economical sequential process [20]. *S. equi* was cultivated in a batch fermentation using HA production medium in 250 mL flasks with 100 mL working volume. After the fermentation process, the cell debris was removed using 0.15% *w*/*v* sodium dodecyl sulfate for 15 min at RT and centrifuged. The supernatant was recovered and passed through a dialysis column with a cellulose membrane of 25 mm × 16 mm and 14,000 typical molecular weight cut-off. Afterwards, the column was incubated in a NaCl-containing solution for 5 days at RT. The dialysate was filtered with a surfactant-free cellulose acetate filter of 0.45 and 0.2 µm and a mixed cellulose esters filter (8 µm). Finally, 96% ethanol was used to precipitate HA in a 1:4 *v*/*v* ratio following centrifugation. The process is illustrated in Figure 3. The HA obtained by the authors was characterized by different instrumental methods, including nuclear magnetic resonance (NMR) coupled with evaluations of its cytotoxicity and bioactivity. In the bioactivity studies performed, it was found that the HA could induce cell proliferation with promising results for cytotoxicity. Although this sequential method was made at laboratory scale and designed to avoid the use of unit operations that are not cost-efficient, like ultrafiltration, the obtained yield of ca. 12 g/L HA showed promising results for the extraction of hyaluronan, as the yield was higher than others reported in the literature. It is important to note that this procedure was performed on a very small scale in a laboratory, but it cannot be guaranteed that the high yield will be maintained when the procedure is performed on a larger scale. In the present work, other studies with high yield and at a larger scale will be presented, without forgetting that each of these works seeks to extract HA with a certain molecular weight needed for a specific application in a product or process, so the scales of process and yield may vary.

**Figure 4:**
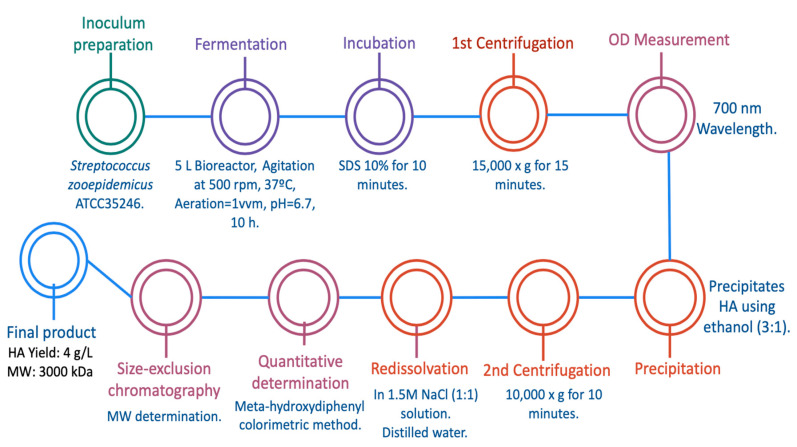
Hyaluronic acid production by *Streptococcus equi subsp. zooepidemicus* ATCC35246 in cheese-whey culture media [21].

Interestingly, four years earlier, Amado et al. worked in a cost-effective alternative for HA production by *Streptococcus equi subsp. zooepidemicus* ATCC 35246 on a larger scale than Güngor et al. using cheese whey as a cost-effective formulated media. This latter aspect becomes relevant when trying to replace synthetic and expensive media based on nitrogen and carbon sources. Amado’s research team used *Streptococcus zooepidemicus* in batch fermentation, using 5 L bioreactors with agitation at 500 rpm and aeration (1 vvm), as graphically described in Figure 4. First, cheese whey culture media was properly formulated with the sugars, salts, and yeast extracts needed, and then the bacteria was cultured in the 5 L bioreactors with a working volume of 4.5 L. The fermentation process occurred over 10 h, controlling the pH at 6.7 and a constant temperature of 37 °C. After the fermentation, samples were incubated with 10% of 5% (*w*/*v*) SDS for 10 min and biomass was removed from the media with an initial centrifugation at 15,000× *g* for 15 min. Then, the optical density (OD) of the supernatant was measured at 700 nm and further subjected to a precipitation step to selectively separate the HA from all the unwanted compounds. This precipitation was performed using ethanol (3:1), a second centrifugation at 10,000× *g* for 10 min, and a redissolution in 1.5 M NaCl (1:1) solution. This selective precipitation was repeated twice, and thereafter HA was dissolved in distilled water. Subsequently, the quantitative determination of HA content was performed by using the colorimetric method of meta-hydroxydiphenyl, and once the concentration was known, the molecular weight was determined by a size-exclusion chromatography using an ultrahydrogel linear column. The final results were positive for the research team, because purified HA was obtained with a yield as high as 4 g/L and a HMW of approximately 3000 kDa. These results demonstrate that a novel alternative such as cheese whey culture medium can produce HA with high yield and HMW. These results were not as good as those presented by Güngor et al., but the HA had the desired characteristics for therapeutic and cosmetic purposes, at a very low cost and with a higher molecular weight compared to the previous work [21].

**Figure 5:**
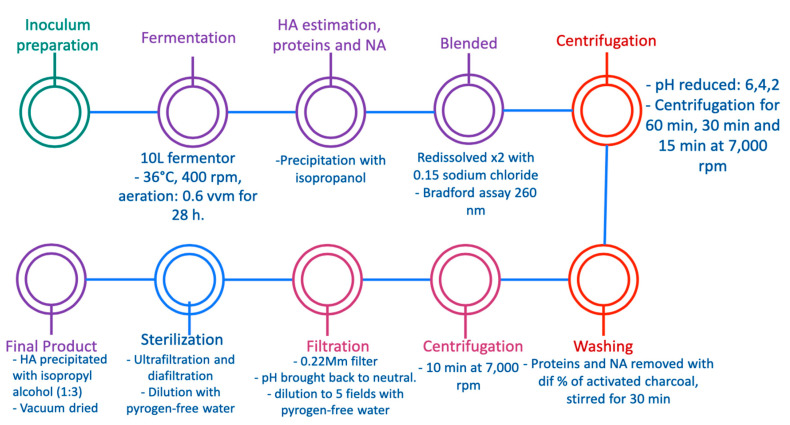
Recovery of hyaluronic acid from viscous culture broth of *Streptococcus equi* subspecies zooepidemicus MTCC 3523 [22].

In 2011, Narala et al. developed an efficient alternative for HA production and extraction from *Streptococcus equi* subspecies zooepidemicus MTCC 3523, which was obtained from the Institute of Microbial Technology, using modified Van de Rejin Kessler medium containing a carbon source and yeast extract. The overall production strategy is described in Figure 5. The researchers used a 10 L fermentor and bacterial growth was allowed at 36 °C with 400 rpm for 28 h and an aeration rate of 0.6 vvm. After the fermentation process, an estimation of proteins and nucleic acids was carried out by precipitation of HA with isopropyl alcohol to avoid the presence of components in the culture medium; then the HA was redissolved twice in 0.15 mM sodium chloride and the estimation of proteins was determined by a Bradford assay by measuring the absorbance at 260 nm in a UV spectrophotometer. For isolation and purification of HA, the pH of the fermented culture broth was reduced to 6.0, 4.0, and 2.0, and the cells were separated by centrifugation for 60 min, 30 min, and 15 min at 7000 rpm, respectively. The supernatant was removed from cell broth by treating with different concentrations of activated charcoal and stirred for 30 min, then centrifugation was carried out at 7000 rpm for 10 min. The pH of the supernatant was brought back to neutral and diluted to 5 fields with pyrogen-free water. A sterilization process of diluted HA solution was carried out by passing it through a 0.22 m filter, and the sterilized HA was further purified via ultrafiltration and diafiltration (DF) mode after dilution with pyrogen-free water using a 300 kDa membrane. the resulting retentate was concentrated to half of its original volume. At last, the concentrated HA was precipitated with isopropyl alcohol (1:3) and vacuum dried. The concentration of HA, proteins, and nucleic acids was monitored in each purification process [22]. The results of the study are comparable with Güngor et al.’s work, both of whom obtained HA at a small scale. Thus, it cannot guarantee maintenance of high quality at a large scale, but it is worth mentioning that Güngor et al.’s method may be more effective as it is more cost effective, and the production of HA yield is higher and less time consuming.

**Figure 6:**
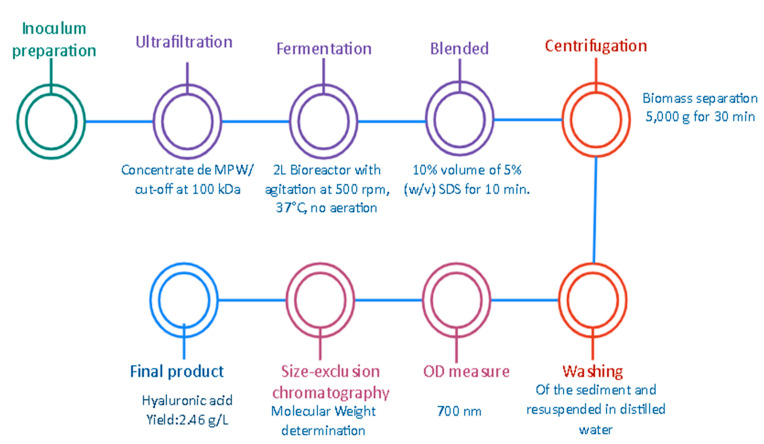
Production of HA by *Streptococcus zooepidemicus* using marine by-product media from mussel-processing wastewaters and tuna peptone viscera [23].

Additionally, Vazquez et al. [23] reported that the production, extraction, and purification of HA by *Streptococcus zooepidemicus* (ATCC 35246) is a viable alternative in order to obtain high MW products that can be used in the pharmaceutical sector or industry. The process involves several stages in order to obtain the desirable product, focused on the optimization of multiple variables and conditions of the fermentation in order to improve the efficiency and the economic viability. It is known that around 80% of the total costs are related to the medium used, specifically the nutrients such as the proteins and sugars that are required. For that reason, the use of an alternative culture media based on marine by-products (MPW) as a sugar source and tuna peptone viscera as a protein source or substrate is proposed. First, the selection of the microorganisms and the preparation of the fermentation broth were developed, in which the strain was stored at −80 °C, the peptones were preliminarily prepared, and the MPW were concentrated by an ultrafiltration using a membrane of 100 KDa (see Figure 6). Then, the batch fermentation was carried out in a 2 L bioreactor using controlled conditions such as pH, temperature, no aeration, and agitation at 500 rpm. The obtained samples were mixed with SDS and then biomass was separated by centrifugation (5000× *g* for 30 min) to obtain a sediment that had to be washed and resuspended. To obtain the HA, a precipitation step using ethanol and centrifugation was required. As a result, a high production of HA of approximately 2500 KDa was obtained with a yield of 2.46 g/L with an improvement in the reduction of the production costs by almost 50%. This aforementioned research proved to be an affordable alternative to implementing the culture media, sugars, and essentials or substrates that are required components in order to obtain the desirable product, presenting lower costs and favorable results. This allows the economic viability of the project to be implemented and the impact of some waste to be reduced, generating a positive impact on the environment.

Other studies have proposed novel methods for a befitting extraction of hyaluronic acid produced by different bacterial species in fermentation processes. All of these studies are presented in Table 3, where the main characteristics of the process, remarks, and yielded results are summarized. For example, Reddy and Kuranakaran [24] described the extraction of HA produced by *Streptococcus zooepidemicus* strain 3523-7 in a fed-batch fermentation with a working volume of 12 L. Since the use of detergents, solvents, and enzymes in extraction processes are not cost effective and often cannot be applied to an industrial scale, the authors strategically proposed an initial purification process with activated charcoal and trichloroacetic acid to remove the cell debris. Afterwards, the clarified HA solution was treated by filtration, ultrafiltration, and precipitation with isopropyl alcohol (1:3 *v*/*v*). The final purity of the product was as high as 99.2% with a yield of 2.3 g/L of clinical grade HA. Likewise, Sousa et al. [25] performed size exclusion chromatography (SEC) for the purification of HA from the same *Streptococcus zooepidemicus* species. Pre-purification steps included centrifugation and several precipitations with ethanol; however, it was found that the efficiency of the purification was reduced with the increasing number of steps, along with the loss of low-molecular-weight fractions of HA. In this research, size exclusion chromatography was able to remove proteic contaminants, yielding 87% purity. However, the yield obtained was way lower compared to the previous work mentioned, alongside the smaller purity percentage, which could be attributed to the advantage of filtration technologies over size exclusion chromatography, but also other factors like the scale of the process and strain characteristics. 

**Table 3:** Advances in extraction and production of HA using different bacterial approaches.

Characteristics of HA	Methods	Process Parameters	Medium Parameters	Yield *	Remarks	Reference
MW: 79 kDa From *Streptococcus equi* ssp. HA of low MW for medical purposes	-Fermentation -Dialysis and sequential filtration -Precipitation	-Dialysis column with cellulose membrane (25 mm × 16 mm, 14.000 molecular weight cut-off) done at RT for 5 days -Filters with a porosity of 8 (surfactant-free cellulose acetate) and 0.45 µm following 0.2 µm (mixed cellulose esters) -Precipitation with ethanol (96% *v*/*v*, 1:4 *v*/*v*)	-Glucose -Peptone -Yeast extract -K_2_SO_4_, MgSO_4_, Na_2_HPO_4_, FeSO_4_, NaCl -Batch fermentation mode (100 mL working volume)	12 g/L	-Production at laboratory scale -Less expensive than using ultrafiltration	[20]
$\mathrm{MW}:\;2.5\times 10^{3}$ kDa From *Streptococcus zooepidemicus* HA for medical purposes	-Fermentation -Filtration followed by ultrafiltration -Precipitation	-Fed-batch fermentation (12 L working volume) -Filtration with 0.45 µm filters (293 mm cassette holder) -Ultrafiltration in diafiltration mode using a 300 kDa cut-off cassette -Precipitation with isopropyl alcohol (1:3 *v*/*v*)	-Chemically defined medium -pH 7.2 ± 0.2 -Temperature 36 °C -Agitation 200–400 rpm	2.3 g/L	-HA with 99.2% purity -Isopropyl alcohol as a cheap option to efficiently remove the final endotoxins	[24]
MW: Varying MW From *Streptococcus thermophilus*	-Fermentation -Centrifugation -Dialysis	-Centrifugation at 18,700× *g* for 30 min -Dialysis against ultrapure water for deionization. Spectra/Por membrane with a molecular mass cutoff of 3500 Da.	−10% skim milk as culture medium -Temperature 42 °C -No agitation	8 × 10^−3^ g/L	-The HA was produced in a wide variety of molecular weights. -The bacteria used were GRAS; therefore, toxins produced were not a problem.	[26]
MW: not specified From *Streptococcus equi RSKK* 679	-Fermentation -Precipitation -Centrifugation -Affinity adsorption	-Precipitation by different amounts of 10% hexadecyltrimethylammonium bromide -D-glucuronic acid (DGA) imprinted particles (100 mg) were treated with HA supernatant (10 mL) for 2 h. -DGA imprinted particles were placed in desorption media for 2 h at 25 °C and 600 rpm. -Desorption carried out with 1 M NaOH	-Working volume 100 mL -Glucose -Yeast extract -Na_2_HPO_4_ -MgSO_4_ -Temperature 37 °C -Agitation 250 rpm -Culture carried out for 20 h	2.3 g/L	-Microbeads showed high adsorption capacity (810 mg/g) and high selectivity for HA. -The process is low cost and compatible with biological systems.	[27]
$\mathrm{MW}:>10\times 10^{3}$ kDa From *Streptococcus zooepidemicus*	-Centrifugation -Precipitation -Size exclusion chromatography	-Precipitation: (1.5:1) ethanol:supernatant (four times) -Size exclusion chromatography in semi-preparative scale -Superose 6 10/300 GL column (300 × 10 mm I.D.) -Injection volume of 250 µL -Room temperature -0.1 M NaNO_3_ as mobile phase	-Synthetic medium -Submerged fermentation	0.78 g/L	-Efficiency of purification is reduced with the increase in pre-purification steps. -SEC was needed to obtain HA free of proteic contaminants for cosmetic and pharmaceutical use. -The precipitation steps helped decrease HA fractions of low molar mass.	[25]
$\mathrm{MW}:\;1.5\times 10^{3}\;$ kDa From *Streptococcus equi subsp. zooepidemicus*	-Fermentation -Diafiltration	-Working volume 4 L -Planar polyethersulfone (PES) membrane, 100 kDa NMWCO -Transmembrane pressure of 2.5 bar -Retentate flow rate 36 mL/min -Room temperature	-Culture carried out for 48 h -Glucose -Tryptone -Yeast extract -MgSO4 -K2HPO4 -KH2PO4 -(NH4)2PO4	0.79 g/L	-Purity reached after 7 diavolumes	[28]
MW: 70 kDa From genetically modified *Corynebacterium glutamicum*	-Fermentation -Centrifugation -Size exclusion chromatography	-Fermentation time of 35 h in 2 L fermentor -Isopropanol 1:1 ratio at −20 °C -Centrifugation at 400 rpm, 30 min -Incubation with active charcoal 1% at 25 °C (1 h). -Centrifugation at 4000 rpm -TCA 100% 1:10 ratio for 30 min on ice -Centrifugation 16,000 rpm, 30 min -Dilution of the supernatant with chloroform-butanol (1:4) and stirring for 30 min -Centrifugation at 7000 rpm, 30 min -SEC in a pre-equilibrated (0.05 mM dihydrogen phosphate buffer, pH 7) Sephadex G100 (1.5 × 25) columns; flow rate of 0.14 mL/min	-CGXII minimal medium pH 7 -(NH_4_)_2_ SO_4_ (5 g/L) -Urea (5 g/L) -K_2_HPO_4_ (1 g/L) -MgSO_4_ (250 mg/L) -CaCl_2_ (10 mg/L) -Temperature 30 °C -Agitation 200 rpm	2.15 g/L	-GRAS microorganism -The process will likely be more efficient if worked with lower temperatures. -It was found that changes in the media parameters strongly affected the MW of HA.	[29]
$\mathrm{MW}:\;\text{\textasciitilde{}}4\times 10^{3}\;$kDa From *Streptococcus zooepidemicus* HA of medical grade	-Precipitation -Centrifugation -Charcoal filtration -Diafiltration -Microfiltration	-10 L fermentor, agitation at 400 rpm, temperature 37 °C, aeration 2 vvm -Precipitation with 2-propanol (1:1 *v*/*v*) -Resuspension with 3% sodium acetate -Silica gel 2% treatment at room temperature, 150 rpm for 2 h -Centrifugation 18,000× *g* for 20 min, 4 °C -Charcoal filter (0.45 µm) -UF in DF mode with a 50 kDa cut-off polyethersulfone cassette -Filtration with a 0.22 µm filter	-Sucrose (20 g/L) -Casein enzyme hydrolysate (25 g/L) -Yeast extract (3.5 g/L) -K_2_HPO_4_ (2 g/L) -NaCl (1.5 g/L) -MgSO_4_ -7H_2_O (0.4 g/L) -Culture carried out for 28 h	5–6 g/L	-An increase in sucrose concentration and a decrease in casein enzyme hydrolyzate resulted in higher HA production. -Complies with the requirements of the British Pharmacopoeia.	[30]
MW: 50 kDa From *Streptococcus zooepidemicus* HA with excellent biocompatibility	-Fermentation -Diafiltration -Purification by adsorbents	-5 L bioreactor with agitation at 300 rpm, 25 h,1.0 vvm of aeration -$\mathrm{Diafiltration}\;\mathrm{cassette}\;0.5\;m^{2}\;$30–50 kDa cut off. 7 diafiltration cycles -Adsorbent 2% *w*/*v* (alumina and activated carbons) into 1000 mL diafiltered broth; filter of 0.45 µm for adsorbent removal -3 L of acetone and stirring for 10 h solidification; RT conditions	-Yeast extract -Potassium phosphate -Glucose (60–80 g/L)-Magnesium sulfate -Sodium chloride -L-glutamate	3.6–3.9 g/L	-The best cut-off cassette for diafiltration was 50 kDa. -Endotoxines were eliminated by using adsorbents. Alumina removed the highest level of endotoxins (99.7%) and activated carbons for the HMW impurities. -HA presented a very good biocompatibility.	[31]
MW: 1 kDa From engineered *Escherichia coli HA03GlcA* HA with excellent biocompatibility	-Genetic engineering for knock-out and knock-in genes -Co-fermentation -Centrifugation -Precipitation	-Incubation of mixture (SDS and cell broth) at RT for 10 min and 200 rpm -1st centrifugation: 13,000 rpm at 4 °C for 10 min; 2nd centrifugation: 5000 rpm for 20 min -Precipitation of the supernatant with 3 volumes of ethanol with overnight incubation at 4 °C	-Ampicillin, kanamycin, and chloramphenicol for selective transformed cells -Luria–Bertani culture media containing mainly yeast extract, peptone, and NaCl	0.03 g/L	-*Eschericia coli* is a endotoxin-free bacteria -Co-fermentation of glucose and galactose -The procedure is expensive because of the genetic-engineering methodology.	[32]
$\mathrm{MW}:\;2.36\times 10^{3}$ kDa From *Streptococcus zooepidemicus* HA-13–06 HA with high MW	-Two-stage fermentation -Carbazole method for HA concentration measurement	-10 L bioreactor, 24 h fermentation, 1 vvm aeration, agitation at 600 nm -1st fermentation: 31 °C, pH 8.0, 10 h -2nd fermentation: 37 °C, pH 7.0, 14 h -Cell OD measurement at 700 nm	-Glucose -Yeast extract -Tryptone -Magnesium sulfate -Dipotassium hydrogen phosphate	4.75 g/L	-Aeration enhanced glucose uptake, increasing HA production. Moderate agitation improved HA yield. -1st fermentation helped with the MW and the 2nd fermentation with the high yield of HA. -pH, aeration, agitation, and temperature were influential factors.	[33]
MW: 429 kDa From *Streptococcus zooepidemicus* SZ042 (*Vhb* expression) HA produced by modifying culture media conditions	-Genetic engineering for expression of the Vhb gene -Batch fermentation -Precipitation -Quantification using the carbazole reagent method	-10 L bioreactor (7 L working volume) -Centrifugation at 10,000× *g* for 20 min -Precipitation of HA using a treatment with ethanol (2:1) -Cooling down at 4 °C for 1 h -Constant temperature of 30 °C and pH 7.2	-Casein hydrolysate -Yeast extract -NaCl -Magnesium sulfate -Sucrose -KH_2_PO_4_ -K_2_SO_4_ -FeSO_4_ -MnSO_4_ -Trace elements (2.5%)	6.7 g/L	-*Vhb* gene expression helped bacteria enhance the carbon source use, producing more HA. -The optimum carbon concentration for maximum HA production was only 30 g/L of sucrose.	[34]
MW: 2.21 × 10^3^ kDa From *Streptococcus equi* subsp. *zooepidemicus* HA of MW for multiple purposes	-Mutation of the strain in order to present a deficiency of β-glucuronidase, using size exclusion chromatography, multi-angle light scattering (SEC/MALS) analysis -Microcentrifugation	-BF (100 mL bioreactor) -Inoculation of 2 mL of THY broth -Microcentrifugation at maximum speed (13,400 rpm)	-Glucose 40 g/L -Tryptone 10 g/L -Yeast extract 2.5 g/L -Anaerobic conditions	0.443 g/L	-The presence of glucuronic acid, as a result of enzymatic degradation of hyaluronic acid, can induce the expression of genes that utilize glucuronic acid.	[35]
$\mathrm{MW}:\;\text{\textasciitilde{}}2.5\times 10^{3}$ kDa From *Streptococcus* *zooepidemicus* HA of high MW for pharmaceutical purposes	-Ultrafiltration -Fermentation -Centrifugation. -Washing Resuspension -Precipitation	-BF (2 L bioreactor) -Agitation at 500 rpm, no aeration, 37 °C, and pH controlled with sterile NaOH (5 M) -Ultrafiltration using membranes with cut-off at 100 kDa -1st centrifugation at 5000× *g* for 30 min (separation of mass) -2nd Centrifugation at 5000× *g* for 10 min	-Sugar source: mussel-processing wastewater (MPW) -Protein substrate: tuna peptone (TP) from viscera residue -Initial pH adjusted to 6.7 -Media was sterilized at 121 °C for 15 min	2.46 g/L	-The use of a marine by-product media achieved a reduction in cost by more than 50%. -Offers an alternative to replace expensive commercial sources of carbohydrates and proteins.	[23]
$\mathrm{MW}:\;5.9\times 10^{3}$ kDa From *Streptococcus* sp. ID9102 (KCTC1139BP) HA for e medical and cosmetic purposes	-Fermentation -Statistical approach -Inoculum -Culture media	-BF of 75 L (pilot scale fermentation) -Fermentation performed at 36 °C, 0.5 vvm, and 400 rpm for 24 h -Statistical analysis was carried out using a Taguchi orthogonal array design. -To evaluate the cell growth the optical density was measured (spectrophotometer).	Medium: -Glucose 40 g/L -Yeast extract 7.5 g/L -Casein peptone 10 g/L -400 rpm -0.5 vvm -pH of the medium adjusted to 7.0 using 0.1 N NaOH	6.94 g/L	-The optimization of medium components using a statistical approach was reported. -Glucose was the best carbon source for HA production by Streptococcus sp. ID9102.	[36]
$\mathrm{MW}:\;1.1\times 10^{3}-1.2\times 10^{3}$ kDa From *Bacillus subtilis* HA for multiple purposes	-Genetic engineering -PCR -Cultivation and fermentation of *Bacillus subtilis*	-Fed BF -Selection of the strain of *Escherichia coli* -Selection of the vector, such as pCR2.1 -Cell removal was done by diluting 1 part culture with 3 parts water, mixing well. -Centrifugation at 30,000× *g* -Cell pellets were washed and dried	-Minimal medium with sucrose as the carbohydrate -Grown in 3 L fermentors -pH of 7 +/− 0.2 at 37 °C -Stirred at 1300 rpm	0.8–1 g/L	-High-quality HA compared to commercially available sources -Maximum production was reached at 25 h into the fermentation.	[37]
MW of HA not specified From *Bacillus subtilis* HA for multiple purposes	-Genetic engineering (selection of the strain and vector, PCR amplification of gene fragments, and construction of the strain) -Cultivation of *Bacillus subtilis* -Recovery of HA by centrifugation	-Bacillus subtilis strain was developed by integrating the HA synthase gene (hasA) and the UDP-glucose dehydrogenase gene of *Streptococcus* (hasB) or of *B. subtilis* itself (tauD) into the amyE locus of the *B. subtilis* chromosome. -PCR for 30 cycles -The transformed strain was grown in LB agar at 30 °C for 16 h -Using a bioreactor for the obtention of HA -Centrifugation 12,000 rpm for 10 min -Precipitation of HA using cetylpyridinium chloride (1.7 w/v)	MMG medium or broth composition per liter: -7.0 g dipotassium phosphate -2.0 g monopotassium phosphate -0.5 g sodium citrate -0.1 g magnesium sulfate-1 g ammonium sulfate -Overnight at 37 °C, stirring at 170 rpm	1.8 g/L	-HA production was achieved by expressing hasA alone, coexpressing hasB or tauD with hasA. -The HA production was enhanced by approximately 200% with the use of a transformed strain.	[38]
$\mathrm{MW}:\;2.09\times 10^{3}$ kDa From *Kluyveromyces lactis* HA for medical purposes	-Genetic engineering -Quantified using high-performance liquid chromatography (HPLC) -SDS, centrifuged, filtration with 0.20 µm filter, deionization, and the carbazole method	-0.1% SDS for uncoupling the HA capsule surrounding the cell wall, centrifuged 6000× *g* 4 °C; then supernatant was filtered with 0.20 µm filter and the HA was purified by washing the medium twice with 3–4 volumes of 100% ethanol. The HA pellet formed was resuspended in 50 mL of deionized water, and the carbazol method was used for HA quantification as previously described.	-Modified YPD medium as previously described. -Yeast extract (7.5 g/L) -Peptone (10 g/L) -Glucose (40 g/L) -K2HPO_4_ (2.5 g/L) -MgSO_4_ (0.9 g/L) -H_2_O -NaCl (5 g/L) -Glutamine (0.4 g/L) -Glutamate (0.6 g/L)	1.89 g/L	- The addition of human hasA genes in the *K. lactis* genome did not result in the synthesis of hyaluronic acid.	[39]
MW: not specified From *Streptococcus equi subspecies zooepidemicus*	-Fermentation -Centrifugal separation of cells from culture broth at low pH	-BF cultures -pH was reduced to 6.0, 4.0, and 2.0 and the cells separated by centrifugation for 60 min, 30 min, and 15 min at 7000 rpm respectively. -Concentration of protein present during purification of HA -> Bradford assay -The HA was precipitated with isopropyl alcohol to avoid interference by the components.	-Carbon source 20 g/L, yeast extract 15 g/L; 1% inoculum and 1% yeast extract -Viz., temperature, pH, and treatment with activated charcoal were included	5.6 g/L	-A simple and efficient method for the separation and recovery of HA from highly viscous culture broth was developed. -The centrifugal separation of cells from culture broth at low pH became much more efficient vs. neutral pH.	[22]
$\mathrm{MW}:\;3.1\pm 0.4\times 10^{3}$ kDa From *Streptococcus equi subsp. zooepidemicus*	-High-pressure liquid chromatography (HPLC)	-BF of S. zooepidemicus was performed under standard conditions. -Range of pH 6.3 to 8.0-Aeration rate of 0.2 vol/vol/min	-30 mL of M17 -Glucose broth -The contents were added to 70 mL of VIG broth and 250 mL of VIG broth in a 500 mL measuring cylinder.	2.7 g/L	-The maximum HA concentration and bacterial specific growth rate were temperature dependent -Aeration resulted in no change in the maximum specific growth rate of microorganisms but enhanced HA production.	[40]
MW: 1 to 10 kDa From *S. equi subsp zooepidemicus* (ATCC) 39920	-Extraction and purificationFermentation, centrifugation, precipitation, size exclusion chromatography, and gel filtration	-Batch culture fermentation at 37 °C, 150 rpm for 24 h -Centrifugation at 3200 rpm -Precipitation 1.5:1 (*v*/*v*) ethanol:supernatant -Polysep-GFC-P6000 column of the gel filtration	-Agricultural resource derivatives for the supplementation of the media (10%, v/v)	0.89 g/L,	-Hydrolysate soy protein concentrate (HSPC) and whey protein concentrate (WPC) media were the most effective for the production of biomass.	[41]
$\mathrm{MW}:\;4\times 10^{4}$ kDa. From *S. equi subsp zooepidemicus* (ATCC 39920)	-Fermentation -Centrifugation -Filtration using membranes and high-performance liquid chromatography	-Batch fermentation (3 L), agitation 250 rpm and aeration 2 vvm -Pore size of 0.2 μm -Centrifugation centrifuged at 3200 rpm during 20 min -Three precipitation and dissolution -Carbazol method	-Glucose (25 g/L) -Yeast extract (60 g/L) -Forced aeration 2 vvm	1.21 g/L	-The initial glucose (IGC) fermentation was independent of the oxygen supply. -The molecular weight was affected by the IGC.	[42]
$\mathrm{MW}:\;600-1\times 10^{3}$ kDa From *Lactococcus lactis* NZ9000	-Genetic engineering -Culture media preparation -Fermentation -Centrifugation and MF -Diafiltration with UF -Adsorbent treatment -HA analysis	-2.4 L bioreactor (1 L working volume) -1 vvm of aeration and 200 rpm agitation -Centrifugation at 10,000 rpm for 20 min RT -MF using 0.45 µm membrane -DF using a polyethersulfone UF membrane cassette NMWCO of 300 kDa -Membrane washing with 0.1 N NaOH -Adsorbent treatment with 1% of activated charcoal for 2–3 h with constant stirring -Precipitation with isopropyl alcohol (1:2)	-Brain heart infusion (5 g/L) -Yeast extract (5 g/L) -Ascorbic acid (5 g/L) -Dipotassium hydrogen phosphate (1.5 g/L) -Potassium dihydrogen phosphate (0.5 g/L) -Magnesium sulfate (0.5 g/L)	0.8–1 g/L	-The bacteria strain suffered a knock out of 3 genes for lactate dehydrogenase expression. -DF was followed by an adsorption step, and both helped to increase HA purity (≈100%). -The use of a higher MW cut off membrane is desirable.	[43]
$\mathrm{MW}:\;1\times 10^{3}$ kDa From *Bacillus subtilis 3NA*	-Genetic engineering -IPTG induction -Fermentation -Centrifugation and MF -1st UF -2nd UF and DF -Precipitation -HA analysis	-Genes from S. zooepidemicus ATCC6580 were used -Fed BF in 3 L bioreactor, temperature of 37 °C, agitation of 1200 rpm, pH 7.0, and aeration of 1 vvm -Induction with IPTG 0.1 mM -Centrifugation at 5000× *g* for 10 min and MF with a hollow fiber unit. -1st ultrafiltration with a 750 kDa unit -2nd ultrafiltration with a 0.1µm MWCO -Diafiltration with pure water -Precipitation with ethanol 2:1 1 h at 4 °C	-LB medium -Glycerol (2.44 g) -Yeast extract (5 g/L) -H_3_PO_4_ (0.082 g) -NH_4_ OH (0.29 g) -MgSO_4_ -7H_2_O -Magnesium sulfate (0.5 g/L)	7 g/L	-Bacteria transformation with HA genes, i.e., *hasA, tuaD, gtaB* and *gcaB* *-B. subtilis* could be a cost-effective and eco-friendly alternative for HA production. -This new process could increase the operating profit of a manufacturing plant by more than 100%.	[44]

HA: hyaluronic acid; RT: room temperature; BF: batch fermentation; MF: microfiltration; UF: ultrafiltration; DF: diafiltration. * Yield: grams of HA per liter of fermentation broth.

Moreover, studies with GRAS microorganisms have also gained attention for the production of toxin-free HA for medical purposes. Izawa et al. [26] obtained HA with varying molecular weights from GRAS bacteria *Streptococcus thermophilus* YIT 2048. The purification of HA was carried out through dialysis with a Spectra/Por membrane with a 3500 Da cut-off, albeit the fermentation conditions require further improvements. Although the process only yielded 0.008 g/L of hyaluronan, it shows the potential of the use of *S. thermophilus* YIT 2048 to produce toxin-free HA if an optimal extraction method is employed. In contrast, other species different from the *Streptococcaceae* family can also be used to produce HA with higher or similar yields to those obtained with Streptococcus, avoiding the production of toxins related to *Streptococcus* species that are not considered GRAS. Karami et al. [29] aimed to produce low-molecular-weight HA with the gram-positive bacteria *Corynebacterium glutamicum*, which was genetically modified to obtain different recombinant strains with HA-producing genes (such as hasA, hasB, hasS, and glmU). The recovery process of the HA consisted of centrifugation, the addition of active charcoal for the removal of cell impurities, and size exclusion chromatography. The SEC was performed in Sephadex G100 1.5 × 25 columns. In general, the authors found that the different combinations of genes in the recombinant strains did not have significant differences in the production of HA with high molecular weight, but the media components strongly affected the molecular weight of the HA produced due to the balance of the metabolic flow. The maximum obtained results were 2.15 g/L HA yield with a molecular weight of 70 kDa. 

Nowadays, specific nanotechnology approaches are being studied for the extraction of HA, such as molecular imprinting. Akdamar et al. [27], for instance, reported the separation of HA from *Streptococcus equi* RSKK 679 by glucuronic acid-imprinted microbeads. Molecular-imprinted polymers can be prepared using a fragment of a protein structure as a template. In this case, the glucuronic acid and N-acetyl glucosamine repetitive units from the HA structure were used. The microbeads showed high selectivity towards HA and could be used several times without a loss of adsorption capacity with a yield of 2.3 g/L. Therefore, the authors showed an alternative to traditional and membrane separation techniques at a laboratory scale, presenting a similar yield to studies reported in the literature for larger-scale experiments (see Table 3). It is important to assess the reproducibility of molecular imprinting on industrial-scale fermentations. 

DF has been demonstrated to be able to offer high yields compared to other unit operations [45,46]. This technology has been used for the recovery of specific target molecules, like dextran, from complex fermentation systems [47]. As an example, Oueslati et al. [28] also worked with *Streptococcus zooepidemicus* to produce HA of more than 90% purity with a molecular weight of 1.5 × 10^6^ Da. The extraction was done by DF with a planar polyethersulfone (PES) membrane with a transmembrane pressure of 2.5 bar at RT. The highest purity level (near 90%) was reached after 7 diavolumes, with a yield higher than 90%. Particularly, the number of diavolumes represented the evolution of the HA’s yield and purity; for example, the purity of the HA was stable beyond 6 diavolumes and a decrease of 20% in the HA production took place after 10 diavolumes. It was also important to regulate the salts present in the mixture for the DF, as salts may change the structure of hyaluronan due to the shielding electrostatic repulsion of the carboxyl groups [48]. In contrast, Rangaswamy and Jain [30] documented a process for the purification of HA from *Streptococcus equi* subsp. zooepidemicus with the purpose of obtaining the highest quality over quantity of HA. The recovery steps were established as follows: precipitation, centrifugation, charcoal filtration, DF, and filtration. A single-solvent precipitation was used with the aim of reducing the use of solvent in other unit operations; in particular, the dilution of the HA in DF with the solvent tends to be done at a low concentration, enhancing the HA quality. The sterile final product was obtained with a 65% yield, obtaining 5–6 g/L HA and a molecular weight of about 4 × 10^6^ Da. When dealing with the processing and recovery of molecules via membrane processes, an important aspect that must be studied is the relevant effect of electrostatic interactions between membrane materials and the solutes [49,50,51]. 

Very recently, purification methods using organic solvents have been considered expensive for large-scale downstream processes. In many laboratory-level HA production studies, organic solvents are used due to the highly efficient precipitation results, but the cost is not suitable for large quantities, so many authors have stated that the best method for large-scale HA purification is not the use of organic solvents (chemical method), but rather membrane technologies such as MF, UF, or DF (physical methods). Tangential flow MF and UF were reported to be useful for the separation of HA from *S. zooepidemicus* culture media. At this point, microfiltration membranes (0.45, 0.20 μm in pore diameter) and ultrafiltration membranes (MWCO 300, 100 kDa) made from polyvinylidene fluoride were implemented in a serial process that not only provided high yield (89%), but also saved water [52]. Electrofiltration (membrane filtration coupled with electrophoresis) as a downstream process for the production of HA has also been studied with the aim of increasing concentration of the final product and reducing the environmental issues that come with traditional techniques. HA extraction using electrofiltration has been demonstrated to increase concentration factors while maintaining the same molecular weight and structure compared to filtration experiments without an electrical field involved [53]. 

Very recently, membrane-based technologies have been pointed out for bioseparation processes where the main objective is to recover functional molecules for industrial applications. Membrane technologies such as MF and UF have advantages over traditional separation processes in terms of non-use of chemical reagents, simplicity of operating conditions (temperature and pressure), preservation of active biological properties, and no risk of contamination due to the non-use of biological agents [51]. The right application of membrane-based technology depends on many factors, such as membrane pore size, membrane material, transmembrane pressure, temperature, feed concentration, and physicochemical properties of the desired molecule. Many authors have discussed that MF represents a pretreatment technique since it is useful for removing undesirable biological compounds, macromolecules, or suspended solids from the raw feed stream (e.g., bacterial broth). UF technology is used for the separation of low-molecular-weight molecules in which the proper pore size (of the weight cut-off) plays an important role in the separation [54]. In the case of HA separation by membrane-based technologies, it is important to correctly determine the final use of the compound, as depending on the HA application, the required MW will be different.

The performance of membrane separation techniques is determined by several factors that can affect the permeate yield, and these factors depend on each other. Initially, the operating parameters are critical to obtain accurate separation and maximize permeate flux; for example, the transmembrane pressure (TMP) has a direct effect on membrane fouling (reversible or irreversible); if the TMP increases, there is a linear increase in permeate flux. Similarly, higher permeate flow is obtained by increasing the process temperature, whereby higher temperatures decrease the viscosity of the feed solution, reducing the resistance to flow and, therefore, requiring lower pumping energy and power. Other important operating parameters are the feed flow rate and hydrodynamic conditions, which promote the particle or solute adhesion to the membrane, causing the formation of a fouling layer that seals the pores and does not allow higher permeate flux to be obtained. Finally, as mentioned above, the size of the membrane pores is also important for optimal filtration conditions; the pores must allow free passage of the desired compound, depending on the size and molecular weight cut-off, considering that the MWCO has to be three to six times smaller than the MW of the desired compound [55]. 

The molecular interactions between membrane solutes are relevant, as is, more importantly, membrane fouling. The interactions between the solutes and the membrane can determine the filtration efficiency, which depends on the physicochemical composition of the compound (i.e., its nature, hydrophobic/hydrophilic interactions, charge, morphology, etc.), but at the same time, the materials and characteristics of the membrane are extremely important in the filtration process, e.g., the membrane must have a low affinity towards the solutes (HA) and good stability with the main solvent [45]. A lack of good interactions between the membrane and the solutes causes the fouling phenomenon. Membrane fouling is the accumulation of solutes/substances/solvents on the membrane surface, and it is the most common issue in membrane-based technologies, as it causes a decrease in permeate flux, resulting in low-performance separation, and therefore it is considered the main drawback of pressure-driven membrane processes [56]. All these factors should be well studied to obtain the best results in the process. According to a large number of studies, membrane technologies are cost-effective strategies for the separation of biocompounds; in particular, an MF treatment followed by a UF process could be a very efficient strategy for HA separation, avoiding costly chemical solvents or a large number of centrifugation cycles.

Nowadays, engineered microorganisms generally recognized as safe (GRAS) play important roles for new alternatives in the production of high-value biocompounds. Rajendran et al. (2016) designed an aqueous two-phase system for the purification of hyaluronic acid produced by a metabolically engineered *Lactococcus lactis* following a fermentation process under specific conditions. A DF step was applied for HA separation using a polyethersulfone UF membrane cassette with an NMWCO of 300 kDa under a range of pressure of 1–0–1.5 bar. Finally, the diafiltered broth was treated with an adsorbent treatment of 1% activated charcoal for 2–3 h with continuous stirring and a filtration with a 0.22 µm cellulose acetate filter. The final results showed a high-purity HA with a MW ranging from 0.6 to 1.8 MDa and a total yield of HA of around 0.8–1.0 g/L [43]. Another example of HA production by GRAS was presented in 2021, when a group of Argentine scientists developed a low-cost and sustainable HA production process using the *Bacillus subtilis* 3NA strain as a producer. This new method proposed a genetic-engineering approach and a combination of membrane and chemical precipitation technologies for improved HA extraction. They used MF with a hollow fiber unit for cell body removal, and then two UFs were used to separate HA impurities. These UFs had a 750 kDa cassette and a 0.1 µm MWCO unit fiber, respectively. The final yield was 7 g/L HA with high purity and a HMW of 1 MDa; both values were the ones sought by the team [44]. The high yield obtained in this work may be due to the fact that in recent years new genetic-engineering approaches have been developed in the production of compounds that do not occur naturally in a microorganism, guaranteeing high yield, quality, and safety. In this case, in addition to genetic engineering, a methodology combining a chemical separation method (precipitation) with physical methods, such as UF and MF, was used. If genetic engineering and the combination of separation methods were applied in several papers, an unprecedented purification yields would be obtained.

As mentioned previously, one of the main negative consequences of bacterial system production of HA is the possibility of the presence of microorganism endotoxins in the final product. To face such an issue, Choi et al. conducted a study in which HA was produced and purified from a natural or non-modified strain of *Streptococcus zooepidemicus*, eliminating the endotoxins and high-molecular-weight impurities by using adsorbents such as alumina and activated carbons. The first one showed the best results for the elimination of endotoxins, whereas activated carbons demonstrated a very effective removal of high molecular proteins and nucleic acids. HA purification was led by a DF process using a cut-off cassette of 50 kDa, obtaining HA with a molecular weight of 50 kDa. The yield of purified HA was approximately 60%, and was calculated by comparing the weight of the dry HA with that of the fermented weight [31]. Compared with other works, Choi et al.’s study showed that the use of adsorbents as purification tools could be very effective in removing bacterial toxins, but the main concern is that adsorbents are too expensive to be applied in large-scale processes, so it can be concluded that these chemicals can be used when the biocompound can only be extracted from a strain with endotoxins and the molecule has a high commercial value.

A breakthrough study was developed by Woo et al. in 2019. It was a work full of genetic engineering in which the authors innovated by using a bacterium that naturally does not produce HA: *Escherichia coli*. The galactose-utilizing Leloir pathway was activated by knocking out the *galR* and *galS* genes encoding the transcriptional repressors. A knock-out modification of zwf and pfkA genes was also performed in order to control the consumption rates of glucose and galactose. Additionally, the *hasA* gene from *Streptococcus zooepidemicus* was introduced for the expression of hyaluronic acid synthase, and the gene clusters *galU-ugd* and *glmS-glmM-glmU* were overexpressed in the *Escherichia coli* in order to promote HA production. This new engineered strain was named *Escherichia coli* HA03GlcA. After the batch fermentation process and the other operation units, a total yield of 29.98 mg/L was extracted and purified. Despite the small amount of HA obtained, other characteristics can be highlighted, such as the low molecular weight of the HA (1 kDa) and the high biocompatibility of the HA obtained [32]. This work showed the advances in biotechnology, helping to broaden our outlook for the production of compounds from microorganisms that do not do so naturally, and although the obtaining of HA was very low, this methodology affirms that day by day new products can be generated from novel processes. In this case, the process obtained low concentrations, but the fermentation and bioseparation parameters can be improved, thus taking advantage of the high efficiency of a microorganism such as *Escherichia coli* and obtaining a high yield of toxin-free HA.

In 2018, Liu et al. performed a two-stage fermentation using *Streptococcus zooepidemicus* HA-13-06 with total fermentation for 24 h. They concluded that aeration and agitation are two factors that play an important role in HA production. For instance, aerobic conditions (aeration) enhance the glucose uptake, promoting a production of HA with higher molecular weight. On the other hand, a moderate agitation of 600 nm demonstrated the improvement of HA yield, concluding that pH, aeration, temperature, and agitation parameters have to be controlled in order to obtain the best results. In this case, Liu et al. obtained a yield of 4.75 g/L of HA with a molecular weight of 2360 kDa [33]. This work highlights the importance of fermentation parameters as a way to obtain HA with desired characteristics, either high or low molecular weight or simply to increase product yield. This finding can be applied in other HA extraction methodologies, specifically in the bacterial fermentation stage.

Genetic engineering has been widely used to improve HA production from bacterial systems. Lu et al. developed a bioprocessing strategy with one of the highest reported yields: approximately 6.7 g/L of purified HA. Expression of the *Vhb* gene in *Streptococcus zooepidemicus* bacteria helped to obtain the high-yield result. Thanks to the expression of the *Vhb* gene, the uptake of the carbon source was improved and, thus, the production of HA. The new genetically modified bacterium was named *Streptococcus zooepidemicus* SZ042. A batch fermentation method was implemented by the working team by using a 10 L bioreactor and modifying the culture media contents, and adding sucrose, casein hydrolysate, magnesium sulfate, etc. All these modifications in the bioprocess led to a final yield of 6.7 g/L HA with HMW of 429 kDa [34]. A high yield of HA was obtained due to the genetic improvement applied to the natural producer bacteria and the increase in carbon uptake. This positive result represents an advance in the extraction and purification of HA. Despite the fact that the working volume was large (ca. 10 L), the molecular weight and yield were quite good compared to other works where smaller fermenters were used. Furthermore, Krahulec and Krahulcová (2005) revealed an improvement in HA production by *Streptococcus equi* subsp. *zooepidemicus* strain, which presents a deficiency of β-glucuronidase (mutated strain) at lab scale. Herein, a 100 mL bioreactor was used to carry out a batch fermentation, achieving a higher production of almost 20% compared with a wild-type strain (yield of 0.4430 g/L) and an increase of 2% of the MW (2.21 × 10^3^ kDa) [35]. This improvement revealed favorable results in terms of obtaining the product of interest since a higher production yield was obtained. It is vital to take into consideration that this procedure was developed at a lab scale; therefore, more research is recommended in order to evaluate this increment in yield.

In an early work, Widner et al. (2005) obtained a yield of 0.8–1 g/L of HA in the production by *Bacillus subtilis*, that encodes a gene (hasA) of *Streptococcus equisimilis.* These authors reported the selection of the bacterial stains, the plasmid vector, the PCR process for the cloning of the genes, the development or construction of the strain, the fermentation, and finally, the obtention of the desired product. As a result, it was reported that the obtained product presented improved characteristics compared to the commercial HA [37]. Chien and Lee (2007) also reported production by the same microorganism (*Bacillus subtilis*) with the integration of some genes, such as hasA, hasB, and tauD, evaluating the impact of the presence of each of these in the production and yield. Here, the authors followed a procedure similar to that of Widner et al., but obtaining a higher yield of 1.8 g/L [38]. Although both research groups used *B. subtilis* as the fermenting bacteria and almost the same methodology, the results were different due to the differences between both strains: The strain used by Widner’s team was more complete since more genes responsible for HA production were introduced.

A statistical approach was achieved and reported by Im et al. (2009) in order to optimize the medium components for the production of high MW HA for cosmetic and pharmaceutical approaches. A two-step optimization was developed in order to select suitable components and a concentration that could represent an implementation. As a result, the components were determined as 4% glucose, 0.75% yeast extract, 0.5% NaCl, and others. The fermentation was developed in a 75 L bioreactor at 36 °C. Consequently, the production by *Streptococcus* sp. ID9102 presented a yield of 6.94 g/L [36]. These authors used a different approach to the traditional one and sought to perform an optimization using statistical tools instead of trial and error. This type of approach allows viable and favorable results to be obtained while remaining economically viable, since it does not require too much experimentation, opening an area of opportunity to search for alternatives and implement experimentation conditions or parameters.

As mentioned previously, the contents of the medium and its conditions are important to optimize the cell growth and production of HA. Armstrong et al. proved the importance of pH and temperature during the fermentation and obtention of HA processes. A batch of *S. zoopidemicus* was fermented under different conditions of temperature and pH. Initial experiments were performed under standard conditions, and the effect of pH was examined, ranging from 6.3 to 8.0, resulting in an optimal pH of 6.7 for biomass growth. Furthermore, the temperature was examined over the range of 32–40 °C, resulting in an optimal growth temperature of 40 °C. The process for the extraction of HA was high-pressure liquid chromatography (HPLC) [40]. 

Nowadays, engineered microorganisms play important roles for new alternatives in the production of high-value biocompounds. Rajendran et al. (2016) designed an aqueous two-phase system for the purification of hyaluronic acid produced by a metabolically engineered Lactococcus lactis, following a fermentation process under specific conditions. A DF step was applied for HA separation using a polyethersulfone UF membrane cassette with an NMWCO of 300 kDa under a range of pressure of 1–0–1.5 bar. Finally, the diafiltered broth was treated with an adsorbent treatment of 1% activated charcoal for 2–3 h with continuous stirring and a filtration with a 0.22 µm cellulose acetate filter. The final results showed a high-purity HA with an MW ranging from 0.6 to 1.8 MDa and a total yield of HA of around 0.8–1.0 g/L [39]. 

Finally, Pires et al. [42] reported on the production of HA from *Streptococcus zooepidemicus*, evaluating the IGC conditions in order to understand the impact. First, the culture medium was developed, and then the culture maintenance and inoculum preparation. Then, for the extraction, membranes were used with a pore size of 0.2 μm, among other methods. A yield of 1.21 g/L was achieved [42]. Pires et al. [41] also reported on the production of HA using the same strain, assessing the impact of agricultural resource derivatives. As a result, a yield of 0.89 g/L was obtained, highlighting the use of cashew apple juice as a possible alternative for use as a culture medium [41].

## 3. Advances in HA Extraction from Animal Sources

Clearly, extensive research has been done and reported by multiple authors to ensure the efficient extraction and purification of HA from bacterial sources. In addition to the existence of producing HA from microorganisms, there are also multiple marine and terrestrial sources, such as animal wastes and by-products (rooster and wattle combs, swine, porcine and bovine cartilage, and fish waste) that have recently gained great attention. These animal wastes pose a serious problem to the environment, especially in the case of fish waste, with around 50% of the tissue being discarded. For this reason, the research and development of new approaches that allow for the extraction of this material, along with reducing the problems and impact on waste management, is vital [9].

Facing this concern, Murado et al. [57] used DF to obtain the desired product with high purity (99.5%) from fish eyeballs without a fermentation method. In this case, a bacterial strain was not required, so this represents an alternative approach that opens up new possibilities and even new challenges. These authors reported on an extraction and purification method from biological materials as an alternative with enhanced characteristics and low costs. A polysulfone membrane was used in order to develop the UF–DF at 35 °C, presenting favorable results. Then, a precipitation using ethanol was used, followed by different steps, such as clarification stages, obtaining a yield of about 6.35 mg/mL [57]. 

Furthermore, Amagai et al. [58] reported on an improved method for HA extraction from tuna fish eyeball (*Thunnus obesus)*. First, dissection in a frozen state was carried out, followed by a filtration and resuspension in order to be able to carry out the mucolysin (ED) extraction method. In order to separate and purify, a dialysis was carried out, obtaining a yield of 10.5 mg hyaluronan from one tuna eyeball. Similarly, Sadhasivam et al. [59] described an enzymatic extraction method using papain in order to obtain high-molecular-weight hyaluronic acid (1365.863 kDa) from the liver of marine stingray (*Aetobatus narinari*), which presents significant anti-proliferation and antioxidant activities. The separation and purification method had an anion exchange chromatography presenting a yield of 6.1 mg HA/g dry weight of tissue. 

Alternatively, terrestrial biomass for the extraction and obtention of HA offers a viable alternative, such as rooster comb (39.8 g/kg) and wattle tissue (17.9 g/kg) via cellulose acetate electrophoresis [60]. Overall, the different sources present advantages and limitations that have to be considered based on the requirements, considering the cost, yield, and environmental impact. 

## 4. Conclusions

This review elucidates the great advances in producing and extracting HA from different sources. To date, it is likely that bacterial fermentation seems to be the most promising pathway to produce high-molecular-weight HA. Even if there are various sources to produce HA, the diversity of bacteria able to synthesize this molecule promotes bigger efforts in this field. When dealing with the main strategies for extraction and purification, the main pre-purification steps include typical unit operations such as centrifugation and several precipitations with polar solvents, whereas UF, DF, and size exclusion chromatography stand out as the most preferred method for the final purification. 

An important research scope proposed to improve yield production of the existing bacterial strains deals with genetic engineering. These approaches have shown potential production yields of HA. For example, *Escherichia coli* HA03GlcA has offered interesting outcomes, producing up to 30 mg/L of HA [32]. When compared with the yields offered by other strains ranging from 2.3 to 7 g/L (see Table 3), this genetically modified strain did not produce a competitive yield of HA, but it was relevant to highlight the interesting biocompatibility that the HA displayed.

As part of the recovery protocols, membrane technology, such as UF and DF, stand out as the preferred techniques for the selective extraction of HA from fermentation broths. In order to extend the application of membrane technologies in this field, researchers may consider different factors influencing the performance of membrane separation techniques, such as operating parameters (transmembrane pressure, feed temperature, feed flow, membrane pore size), molecular interactions between membrane solutes, and, more importantly, prominent membrane fouling [54,61]. This latter aspect represents one of the main drawbacks of membrane technologies for long-term operation [56,62]. Therefore, new researchers in the field find in this point a potential research gap to further investigate and implement membrane processes in pilot and large-scale approaches. 

## Figures and Tables

**Table 1:** Advantages and disadvantages of different HA extraction sources [7].

Source	Advantage	Disadvantage
Animal	High yield of HA, products with HMW, natural products	RE or RC, EPP, variation in product quality
Bacterial	High yield of HA, CP, low cost and short time, uniformity of HA and HA with HMW	Possible BE, use of GMOs, RC during production
Yeast	Low-cost production	Dangerous for humans, only one microorganism can produce it.

BE: bacterial endotoxins; GMOs: genetically modified organisms; RC: risk of contamination; RE: risk of endotoxins; EPP: extensive purification process; CP: controlled parameters.

**Table 2:** Applications and required molecular weights of HA.

	Use	Molecular Weight	Reference
Medical	Bone disorders: injections for pain relief. Ophthalmologists: protect and lubricate eyes for the treatment of dry eye. Scaffolds for tissue engineering. Nanoparticles for treatment of inflammatory diseases/active tumor targeting/drug carriers.	Low and high 1400 ± 200 kDa 100 ± 20 kDa Low and High ~200 kDa 2 × 10^3^ kDa High MW Low and high	[10] [11] [12] [13,14,15]
Cosmetic	Skin care: anti-wrinkle, anti-nasolabial fold, anti-aging, and face rejuvenating properties. Dermal filler to lift rhytides and improve facial appearance.	Low MW 50 kDa Monophasic: mixture of high and low MW Biphasic: high MW ~1 × 10^3^ kDa	[16] [17]
Pharmacology	Anti-inflammatory, wound healing, and tissue regeneration.	High MW: 1 × 10^3^–1.5 × 10^3^ kDa	[18]

## Data Availability

Data are contained within the article.
